# Immune Dysfunction as a Cause and Consequence of Malnutrition

**DOI:** 10.1016/j.it.2016.04.003

**Published:** 2016-06

**Authors:** Claire D. Bourke, James A. Berkley, Andrew J. Prendergast

**Affiliations:** 1 Centre for Genomics and Child Health, Blizard Institute, Queen Mary University of London, London, UK; 2 Kenya Medical Research Institute (KEMRI)–Wellcome Trust Collaborative Research Programme, Centre for Geographic Medicine Research, Kifili, Kenya; 3 Centre for Tropical Medicine and Global Health, University of Oxford, Oxford, UK; 4 Zvitambo Institute for Maternal and Child Health Research, Harare, Zimbabwe

**Keywords:** malnutrition, immunodeficiency, inflammation, infection, enteropathy, metabolism

## Abstract

Malnutrition, which encompasses under- and overnutrition, is responsible for an enormous morbidity and mortality burden globally. Malnutrition results from disordered nutrient assimilation but is also characterized by recurrent infections and chronic inflammation, implying an underlying immune defect. Defects emerge before birth via modifications in the immunoepigenome of malnourished parents, and these may contribute to intergenerational cycles of malnutrition. This review summarizes key recent studies from experimental animals, *in vitro* models, and human cohorts, and proposes that immune dysfunction is both a cause and a consequence of malnutrition. Focusing on childhood undernutrition, we highlight gaps in current understanding of immune dysfunction in malnutrition, with a view to therapeutically targeting immune pathways as a novel means to reduce morbidity and mortality.

## Trends

Undernourished children principally die of common infections, and immune defects are consistently demonstrated in under- and overnutrition.

Parental malnutrition leads to epigenetic modifications of infant immune and metabolic genes.

Healthy gut development relies on sensing of dietary nutrients, commensal, and pathogenic microbes via immune receptors.

Recurrent infections, chronic inflammation, and enteropathy compound clinical malnutrition by altering gut structure and function.

Immune cell activation and systemic proinflammatory mediator levels are increased in malnutrition.

Malnutrition impairs immune priming by DC and monocytes, and impairs effector memory T cell function.

Immune dysfunction can directly drive pathological processes in malnutrition, including malabsorption, increased metabolic demand, dysregulation of the growth hormone and HPA axes, and greater susceptibility to infection.

## Malnutrition as an Immunodeficiency Syndrome

Malnutrition, which encompasses both under- and overnutrition, is responsible for an enormous health burden globally 1, 2 (Box 1). Although broadly defined as impaired nutrient assimilation, malnutrition does not simply arise from inadequate food intake. Obesity can develop independently of poor diet and persist despite switching to a healthy diet 3, 4, 5, 6, 7, and stunting prevalence is only modestly reduced by intensive feeding interventions [8]. Despite manifesting as distinct physical defects, several observations implicate shared etiological pathways in under- and overnutrition: early-life undernutrition increases the risk of obesity in later life 4, 9; altered metabolism 10, 11, 12, 13, chronic inflammation 11, 14, 15, and gut dysfunction (enteropathy) 11, 12, 16 are features of both conditions; and excess energy and macronutrient intake is often coincident with micronutrient deficiencies in overweight individuals 17, 18. There is a growing appreciation that malnutrition is complex, reflecting a suite of overlapping comorbidities that are poorly understood 19, 20, 21. Characterizing pathogenesis across the spectrum of malnutrition is essential to underpin novel therapeutic approaches to support international goals to improve nutrition, health, and well-being (https://sustainabledevelopment.un.org).

**Figure 1:**
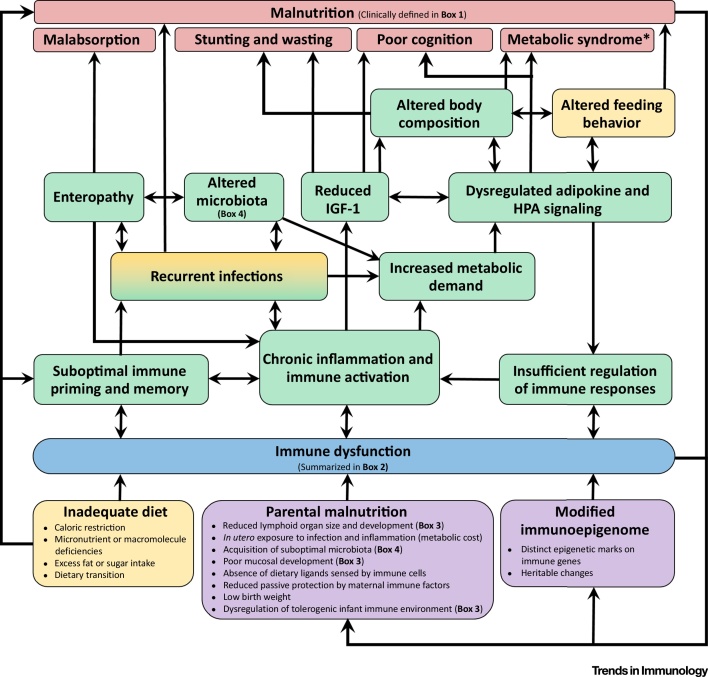
Conceptual Framework for Immune Dysfunction as a Cause and Consequence of Malnutrition. Immune dysfunction can arise before birth via developmental pathways (purple), compounded by environmental and behavioral factors (yellow), particularly those experienced during early life. Immune dysfunction (blue; as defined in a recent systematic review [23] and summarized in Box 2) can contribute both directly and indirectly to a range of causal pathways (green) that lead to clinical malnutrition (red; refer to Box 1 for the clinical features of under- and overnutrition in humans). Abbreviations: HPA, hypothalamus–pituitary–adrenal axis; IGF-1, insulin-like growth factor 1; *, refers to predisposition to metabolic syndrome in adulthood following exposure to undernutrition in infancy.

Undernourished children principally die of common infections 22, 23, implying that mortality is related to underlying immunodeficiency, even in mild forms of undernutrition [24]. Infections are more common and more severe in people with obesity [25]. Defects in both the innate and adaptive arms of the immune system have been consistently demonstrated in undernourished children (Box 2) [23]. In this review we explore the hypothesis that immune dysfunction is both a cause and consequence of malnutrition, and summarize key recent evidence from experimental animal models, human cohorts, and *in vitro* studies. We regard malnutrition as a syndrome in which multiple underlying processes are the cause of elevated mortality and morbidity [20] (Box 1); immune dysfunction is involved in many of these pathways and is therefore a key driver of the vicious cycle that leads to clinical malnutrition (Figure 1). Our focus is childhood undernutrition in developing countries, where the greatest burden of mortality is concentrated [2], but we also identify relevant studies of overnutrition. Throughout the review we highlight research gaps that need to be addressed in future studies and speculate on the potential for immune-targeted therapies to reduce morbidity and mortality in undernourished children.

## Immune Development in Malnutrition

The trajectory of infant immune development during the first 1000 days of life (Box 3) is sensitive to nutritional status, such that impaired immune organ growth 26, 27, 28 and thymic atrophy 23, 29 can be evident at birth in undernourished infants. In a rural Bangladeshi cohort, thymic index (TI) at birth was positively associated with birthweight [27] and all-cause mortality at 8 weeks [26]. Compared to adequately nourished rat pups, pups of dams exposed to protein energy malnutrition (PEM) during lactation have smaller thymuses 30, 31, increased thymocyte apoptosis [31], and a greater proinflammatory thymocyte response to leptin as a result of higher leptin receptor expression [30]. These observations suggest an interacting relationship between nutrition, growth, and immune development, of which thymic size is one indicator. Infant infections, microbial colonization of the gut, T cell activation, and TI have also been linked to breast-feeding practices (Box 3), but the specific breast milk components responsible have not been identified.

It is increasingly apparent that malnutrition can influence immune development before conception because maternal malnutrition confers epigenetic modifications in her offspring 32, 33, 34. Gambian women who conceived during periods of low food availability had lower plasma concentrations of methyl-donor pathway substrates relative to women who conceived during periods of higher food availability, and their infants had distinct percentages of methylation of known metastable epialleles at 2–8 months of age [34]. A randomized, double-blind, placebo-controlled trial of pre- and periconception multiple micronutrient supplementation also found differences in infant DNA methylation between the supplemented and unsupplemented groups 32, 33. Methylation differences in immune (*SIGLEC5*, *CD4*, and *KLRC2*) and innate defense (*BPIL1*, *CHIT1*, and *DEFB123*) genes were evident at birth, and some were still detectable 9 months later [33], suggesting that maternal micronutrient supplementation had a lasting impact on the infant immune epigenome. Distinct methylation of the *IL10* locus has also been identified in Dutch adults exposed to famine *in utero* relative to their unexposed sex-matched siblings over 50 years later [35]. How the perinatal immune epigenome affects long-term immune function has not been assessed; however, the heritable impact of malnutrition suggests that the optimal timing for therapeutic interventions to rescue infant growth and development may need to be re-evaluated 20, 29.

The intimate association between the nutritional status of infants and their mothers means that the relative impacts of immune dysfunction and maternal diet on infant malnutrition are difficult to extricate. However, paternal malnutrition has recently been shown to impart heritable changes on infant metabolism and immune function without *in utero* exposure to a marginal diet 36, 37. Male mice exposed to *in utero* PEM had distinct epigenetic marks at the *Lxra* locus that were inherited by their adequately nourished offspring [37]. *Lxra* encodes a nuclear receptor involved in inflammation and lipid metabolism, and *Lxra*-dependent changes in liver lipid-synthesis genes were evident in second-generation offspring [37]. The sperm epigenome of obese men has also been shown to respond to weight loss after bariatric surgery [36].

## Gut Immune Responses in Malnutrition

The gut is the primary interface between diet and the immune system, and a range of postnatal cues from the microbiota, pathogens, and dietary components are required for healthy development of gut-associated lymphoid tissue (GALT; Box 3).

### Direct Nutrient Sensing

A range of micronutrients and nutrient metabolites act as direct immune stimuli [38], but isolating their independent effects has been largely restricted to murine models in which diet can be carefully controlled. The aryl hydrocarbon receptor (AhR), which binds to metabolites of cruciferous vegetables, is abundantly expressed on murine intraepithelial lymphocytes (IEL; TCRγδ^+^CD44^int^CD25^−^CD69^+^CCR6^−^) and intrinsic AhR signaling is essential for their localization in the gut and skin [39]. Lymphoid tissue-inducer cells (a type of innate lymphoid cell, ILC3, involved in lymphoid development) express AhR and retinoic acid receptor (RAR)-related orphan receptor (ROR) γt, which interacts with the vitamin A metabolite retinoic acid, demonstrating a mechanistic link between nutrient sensing and immune development 39, 40, 41. Murine dendritic cell (DC) subsets vary in their relative expression of retinoid and rexinoid receptor isoforms, leading to selective loss of splenic CD11b^+^CD8α^−^Esam^high^ DC and the associated gut-homing CD11b^+^CD103^+^ DC subset in vitamin A-deficient mice [40]. An analogous population of CD103^+^ DC has been isolated from human mesenteric lymph nodes [42], although their micronutrient receptor profiles and functions have not been investigated in malnutrition. DCs can also synthesize retinoic acid, which influences subsequent T cell trafficking (reviewed in [43]) and promotes T regulatory cell (Treg) induction in the lamina propria (reviewed in [44]). Peyers patch follicular DCs, a specialized cell type promoting high-affinity antibody responses, also express RARs and Toll-like receptors (TLRs) in mice [45]. Both vitamin A and MyD88 deficiencies result in reduced IgA production in murine Peyers patch germinal centers and lower B cell chemoattractant CXCL13 and B cell activating factor expression [45], implicating sensing of both micronutrients and bacteria in mucosal B cell function.

Importantly, nutrient-sensing pathways such as AhR and RAR signaling, directly influence clearance of gastrointestinal infections in murine models 39, 41. Direct nutrient sensing may also enable the gut immune system to adapt to adverse environmental conditions, including micronutrient deficiencies. For example, mice subjected to vitamin A deficiency exhibit profound reductions in ILC3 and antibacterial responses, with a compensatory expansion in IL-13-producing ILC2, leading to increased anti-helminth responses [41]. Collectively these experiments refute the idea that undernutrition leads to a generalized reduction in immune responsiveness, supporting instead a model of phenotypic plasticity in mucosal immunity that responds to nutrient availability. These murine models highlight mechanisms that may be pertinent to human malnutrition because vitamin A deficiency is one of the most common micronutrient deficiencies globally. Meta-analysis of 43 clinical trials of oral vitamin A supplementation in infancy demonstrated a consistent reduction in diarrheal incidence and mortality [46]; however, no trials assessed mucosal immune responses. All-*trans* retinoic acid supplementation led to slight elevations in lipopolysaccharide (LPS)- and vaccine peptide-specific IgA in the whole gut lavage fluid, but not the serum, of Zambian adults in a small typhoid vaccination study 43, 47, highlighting the benefit of investigating both peripheral and mucosal immune responses in future studies.

### Microbiota

In addition to nutrient sensing, microbiota sensing via pathogen-recognition receptors (PRR) is also required for GALT development [48] (Box 3). The configuration of commensal microorganisms (microbiota) detectable in feces is altered in malnutrition (Box 4), and fecal transplants from undernourished children recapitulate weight loss in gnotobiotic mice [13], suggesting that the microbiota may contribute to malnutrition [49]. The immune system has been implicated in this relationship by IgA profiling studies demonstrating that a portion of the fecal microbiota from Malawian infants with SAM is directly bound by mucosal antibodies [50]. Importantly, the IgA-targeted consortia transferred enteropathy to adult germ-free mice, but the bacterial species less often targeted by IgA did not [50]. Pathological changes in the microbiota and nutrient metabolite levels in overweight adults were associated with increased epithelial proliferation rates, IEL numbers, and CD68^+^ macrophages in colonic biopsies [12]. Notably, the 16S ribosomal RNA-detectable fecal community analyzed in these studies represents only a subset of the microbial load present in the gut. Future studies incorporating less-accessible microbes and immune cells from gut tissue will be necessary to delineate their relative roles in undernutrition.

### Infections, Enteropathy, and Inflammation

Healthy gut function requires a large surface area for nutrient absorption, made possible by the complex villous architecture of the intestinal epithelium, and an intact intestinal barrier to prevent pathogen translocation into extraintestinal tissues. Both are markedly impaired in malnourished individuals 51, 52, 53, and there is an almost universal abnormality of gut structure and function among individuals inhabiting impoverished conditions, termed environmental enteric dysfunction (EED) 19, 51, 52. Repeated exposure to enteric pathogens is hypothesized to be the predominant cause of EED in conditions of poor sanitation 20, 51, 52, but multiple causes of enteropathy are likely in developing countries [51]. Microarray of fecal samples from Malawian children with EED identified a range of mRNA transcripts for immune genes significantly correlated with intestinal permeability (percentage lactulose recovery following a dual-sugar absorption test), an indicator of EED severity [54]. These findings implicate immune pathways in gut dysfunction, and future studies will be necessary to explore their relationship with malabsorption, particularly in children with SAM who were excluded from microarray analysis [54].

EED may affect the immune system through several mechanisms: (i) altered nutrient-sensing pathways required for GALT development, (ii) mechanical gut tissue damage releasing host-derived immune-activating damage-associated molecular patterns (DAMPs) and upregulating epithelial repair [54], and (iii) loss of gut barrier function leading to systemic leakage of microbes and pathogen-associated molecular patterns (PAMPs) from the gut lumen (a process termed microbial translocation). Few studies have examined human gut biopsy samples, leaving these overlapping mechanisms poorly distinguished. However, gut damage is evident early in life in developing countries, and incremental acquisition of enteric infections [55], circulating PAMPs, and systemic inflammation are linked to poor linear growth 14, 15, 56. Proinflammatory mediators may contribute to stunting by dysregulating growth hormone signaling, consistent with murine models showing that genetic overexpression of IL-6 negatively regulates insulin-like growth factor-1 (IGF-1) levels [57]. Zimbabwean infants who became stunted by 18 months of age had significantly higher plasma concentrations of proinflammatory markers (C-reactive protein, CRP, and α1-acid glycoprotein, AGP) and lower plasma levels of IGF-1 than their non-stunted counterparts [14]. IGF-1 levels negatively correlated with CRP, AGP, IL-6 and soluble CD14 in this cohort [14], and with CRP, IFN-γ, IL-1α, and MIP-1α in a smaller cohort of Kenyan infants with SAM, stunting, and chronic inflammation [56]. Plasma LPS levels were negatively associated with linear growth in the latter study [56]. Dysregulation of growth factor signaling is also evident in type 1 diabetic enteropathy where high circulating IGF-1 binding protein levels impair the *in vitro* growth and differentiation of IGF-1 receptor^+^ colonic stem cells [58]; thus chronic inflammation in undernutrition could plausibly exacerbate enteropathy through simultaneous epithelial damage and dysregulation of IGF-1-dependent stem cell-mediated mucosal repair.

A better understanding of immunopathogenesis in malnutrition has arisen from a murine model of EED [59]. C57BL/6 mice fed fat- and protein-reduced diets developed mild stunting and wasting and, consistent with observations in human EED 15, 51, 52, poor growth was accompanied by reduced gut integrity and an altered microbiota [59]. The EED gut had more duodenal γδ IELs, higher jejunal proinflammatory cytokine responses to oral doses of bacteria, and higher jejunal and cecal *Salmonella typhimurium* loads post-challenge than adequately nourished animals [59]. Collectively, this model demonstrates that infection-driven dysregulation of mucosal immune function can cause EED, systemic inflammation, and growth failure. Alternative murine models provide proof-of-concept that chronic immune activation, as seen in EED, can drive wasting and infection susceptibility independently of dietary deficiency. Transgenic mice constitutively expressing the activation-induced costimulatory ligand CD70 exhibited progressive CD27-dependent expansion of T effector memory cells (T_EM_) and reciprocal depletion of naïve T cells in secondary lymphoid organs, resulting in weight loss and premature death from opportunistic *Pneumocystis carinii* infection [60]. The CD70–CD27 pathway is postulated to be overactive in human HIV [60] and may also compound infectious mortality in undernutrition.

## Immunometabolic Signatures of Malnutrition

Immunometabolism refers to the chemical reactions required for immune function, and the reciprocal effects of metabolic products on immune cells [61]. Cell activation results in a metabolic shift to meet the high energy requirements of anabolism (*de novo* molecule synthesis) and energy generation via catabolism [61]. Cytokine signaling and T cell receptor engagement can trigger upregulation of amino acid, iron, and glucose transporters to fuel the increased metabolic demands of activated immune cells (reviewed in 62, 63). Immunometabolism has emerged as an important mechanistic pathway in malnutrition from observations that altered energy usage in obesity and metabolic syndromes drives immune activation [6], and that systemic proinflammatory cytokines are elevated together with free fatty acids and ketones in SAM [10]. Inflammation is reduced in children with SAM following therapeutic feeding [10], and short-term shifts in fecal and urinary metabolites occur following dietary alterations in under- and overnutrition 11, 13. Chronic immune activation in malnutrition therefore appears to place high demands on nutrient metabolism, which are likely intensified by recurrent infection, microbiota perturbations, and enteropathy (Figure 1).

Specific nutrient deficiencies may influence T cell metabolism via cytoplasmic nutrient sensors including the glucose sensor AMP-activated protein kinase (AMPKα1), which regulates cell survival post-activation, and the mammalian target of rapamycin serine/threonine kinase complex (mTORc1) 62, 63. Neither AMPKα1 nor mTORc1 activity has been assessed in T cells from undernourished children; however, both sensors can influence T_EM_ maintenance (reviewed by [63]), which is impaired in murine PEM [64]. Malnutrition also alters levels of energy homeostasis mediators, including glucocorticoid hormones of the hypothalamic–pituitary–adrenal (HPA) axis [65] and adipokines released predominantly from adipose tissue 6, 7. Glucocorticoid hormones regulate inflammation and promote thymic and neurocognitive development [65], which are impaired in undernourished children (Box 1). Glucocorticoids are also implicated in obesity because they simultaneously affect adipocyte metabolism and proinflammatory immune mediators. For example, human adipose tissue treated *in vitro* with dexamethasone (a glucocorticoid) upregulates genes associated with lipid, carbohydrate, and amino acid metabolism, alongside leptin and acute phase response genes, but downregulates IL-6, IL-8, and MCP-1, compared to untreated tissue [66]. Despite marked changes in adipose tissue composition, no studies have investigated the relationship between the HPA axis and inflammation in undernutrition.

Of the adipokines, leptin is the most extensively studied because it transmits signals directly to the immune system through leukocyte leptin receptors 30, 67 and delivers feedback signals to the HPA axis via mTORc1 activation to indicate satiety 7, 67, 68. Ugandan children hospitalized for SAM had higher serum leptin levels following nutritional rehabilitation, which coincided with increased insulin and IGF-1, and decreased proinflammatory cytokines [10]. Infants who survived hospitalization had significantly higher baseline leptin levels than those who died [10], highlighting the potential importance of leptin signaling for survival in undernutrition. Mutations in the leptin signaling pathway are risk factors for human obesity 7, 67, and homozygous leptin or leptin receptor deficiency results in excessive eating, early-onset obesity, and an elevated risk of childhood infections, that occur in parallel with T cell hyporesponsiveness, low cytokine production, and reduced CD4:CD8 T cell ratios (reviewed in [67]). High-fat diets have been shown to block mTORc1 activation by leptin in the hypothalamus, which may explain continued hyperphagia in overnutrition [68].

One hypothesis for the paradoxical link between early-life undernutrition and obesity in adulthood (Box 1) is that broad metabolic trajectories become fixed in infancy to reflect environmental conditions at the time, but can lead to subsequent metabolic maladaptation if the adult environment changes [4]. Observations from the Dutch Hunger Winter of 1944–1945 corroborate this hypothesis because infants of mothers exposed to the famine had a higher risk of glucose intolerance, coronary disease, and obesity than their unexposed siblings, despite returning to a pre-famine diet after 1945 [9]. Similar to epigenetic programming of infant immunodeficiency by parental malnutrition, the risk of metabolic syndrome may be epigenetically programmed. For example, maternal malnutrition during the Dutch Hunger Winter modified the infant *IL10* locus as well as genes associated with growth hormones, cholesterol transport, lipid metabolism, and the HPA axis that persisted decades later [35]. Infant gender and CpG methylation of the retinoid-X receptor α (*RXRA*) and endothelial nitric oxide synthase (*NOS3*) genes explained more than 25% of the variation in childhood adiposity in healthy British 9-year-olds, and *RXRA* methylation was higher in infants whose mothers had the lowest carbohydrate intake during early pregnancy [5]. Heritable immunometabolic changes during malnutrition could provide a basis for the intergenerational cycle of stunting evident in developing countries (Box 1) [20]. This hypothesis was recently explored by subjecting 50 generations of Wistar rats to PEM and micronutrient deficiency, which resulted in animals with low birthweight and stunting followed by elevated adiposity and insulin resistance relative to their *ad libitum*-fed counterparts [69]. Following two generations of *ad libitum* feeding, rats from the undernourished line had normal birthweight but retained an enteropathy and metabolic syndrome-prone phenotype related to histone modification of the insulin 2 (*Ins2*) promoter [69]. Thus the predisposition to gastrointestinal, growth, and metabolic defects associated with malnutrition may be imprinted far earlier than previously assumed 28, 70.

## Immune Priming and Memory Responses

Infectious deaths in malnourished children could relate to diminished immunological memory responses to common pathogens. Some studies have identified reduced vaccine-specific antibody titers and seroconversion rates in children with SAM 23, 71, 72, but there is limited evidence for reduced vaccine efficacy in malnutrition 71, 72, and few studies have investigated immunological memory in malnutrition beyond assays of antibody quantity 23, 71, 72.

An unexplored mechanism that may be relevant to human malnutrition is homeostatic memory cell maintenance because protection from reinfection relies on antigen-independent persistence of memory T and B cells primed to respond rapidly upon re-challenge. In PEM mice, proportions of infection-induced lymphocytic choriomeningitis virus (LCMV)-specific CD44^+^CD8^+^ T_EM_ were reduced, and T_EM_ adoptively transferred from protein-sufficient animals were not maintained [64]. T_EM_ produced less IFN-γ upon LCMV peptide restimulation, and had impaired proliferative responses and higher viral loads post-challenge, indicative of functionally impaired immunological memory to LCMV [64]. In a separate study, PEM mice had more *Mycobacterium tuberculosis* bacilli in their lungs and reduced clearance post-vaccination compared to protein-sufficient mice, which was attributed to reduced lung CD4^+^ T cell IFN-γ, TNF-α, and IL-2 responses to mycobacterial antigens [73]. It is promising that, in both the LCMV and tuberculosis models, improved diet led to reconstitution of memory T cell responses and improved pathogen clearance in malnourished animals 64, 73.

The capacity for innate immune cells to generate immunological memory suggests that immunology studies of human malnutrition should extend beyond antibody titers and memory lymphocytes. Trained immunity is a collective term for the memory-like responses of innate immune cells that mediate enhanced protection against secondary and heterologous infections following a primary T and B cell-independent stimulus [74]. Human peripheral blood mononuclear cells and purified monocytes exposed to *Candida albicans* for 24 h *in vitro* have enhanced TNF-α and IL-6 responses to restimulation with LPS, poly I:C, *C. albicans*, or *M. tuberculosis* compared to unprimed cells [75]. These changes in innate cell function, also seen in response to BCG vaccination 74, 76, are related to epigenetic reprogramming of monocytes and changes in their PRR repertoire [75]. PRR and nutrient receptor expression have not been evaluated in malnutrition [23]; however, *in vitro* studies of healthy human cells have begun to investigate dietary micronutrient effects on trained immunity. Increasing concentrations of vitamin A cause progressive reduction in TNF-α and IL-6 production levels, reduced TNF-α, IL-1RA, IL-8, and IL-10 mRNA transcripts, increased methyl transferase expression, and an altered histone modification profile in the cytokine promoter regions of LPS-restimulated BCG-trained monocytes [76]. Vitamin A effects on BCG-trained cells could be reversed by inhibiting SUV39H2 [76], identifying a specific methyltransferase pathway that may be dysregulated by vitamin A deficiency.

Innate immune cells are also affected by excessive antigen exposure, which reduces their responsiveness to subsequent stimulation and ability to prime adaptive immune responses. Immunoparalysis describes the immunosuppressed state that follows acute systemic exposure to proinflammatory stimuli, and this may explain the high rate of secondary infections following sepsis [77]. Experimental LPS treatment of humans leads to reduced LPS-specific cytokine responses, lower surface expression of major histocompatibility complex molecules (HLA-DR) by monocytes, and impaired T cell priming [77]. Given that sepsis is a major cause of death in children with SAM [78], potentially arising from microbial translocation [51], immunoparalysis may compound immunodeficiency in malnutrition. For example, a study of Zambian children hospitalized for SAM found a negative correlation between T cell proliferation and plasma LPS levels that was related to DC priming defects [79]. These children had a lower percentage of myeloid DCs, lower spontaneous DC IL-12 production, and fewer IFN-γ^+^ T cells following tuberculin stimulation *in vitro* before compared to after nutritional rehabilitation [79]. Of the cohort, 17% also exhibited impaired DC maturation (lower HLA-DR upregulation in response to LPS stimulation *in vitro*), which was associated with low T cell proliferation [79]. Therapeutic feeding restored DC numbers, cytokine production, and maturation defects; however, infants with low DC numbers at admission were less likely to survive than those with abundant DC [79].

## Prognostic Value of Immune Biomarkers for Malnutrition

The evidence discussed in this review suggests that evaluating the prognostic value of immune biomarkers for malnutrition is warranted. Generating reliable indices of immune function has been limited to date because most studies have focused on cross-sectional cohorts of acutely unwell children [23] in whom the relative effects of infection and nutrition cannot be discriminated. Incorporation of longitudinal immune assessment into ongoing randomized controlled trials of water/sanitation/hygiene (WASH) and nutritional interventions in developing countries will be necessary to characterize the nature, timing, and extent of immune dysfunction and the impact of public health interventions in malnourished infants (e.g., SHINE [80], WASH Benefits [81], and MAL-ED [82]). Such studies are also necessary in milder forms of malnutrition that are associated with an elevated risk of infectious mortality [24]. For example, stunting affects almost one-third of children in developing countries, and there are more deaths from pneumonia and diarrhea globally among apparently healthy children with stunting than among hospitalized children with SAM [20]. Ethical and logistical constraints on invasive tissue sampling have limited biomarker identification of immune dysfunction in gut and adipose tissue. Efforts are currently being made to define tissue-specific markers of immune function that can be assayed in non-invasive biological samples such as urine, stool, blood, and saliva 15, 82, including cross-validation of enteropathy biomarkers with direct visualization of the gut by confocal endomicroscopy [53].

Perhaps as a result of these limitations, associations between individual immune parameters and malnutrition have been relatively weak to date, compromising their usefulness as biomarkers to guide clinical interventions. Multiple immune genes are implicated in EED [54], and immune defects in undernutrition are wide-ranging (Box 2), highlighting that a single pathway is unlikely to explain the immunopathology of malnutrition. Integrating multiple biomarkers into a single index can more effectively distinguish between malnourished and adequately nourished children than individual analytes 15, 16, and such statistical approaches could be readily adapted to immunological data. We propose that an immune function-for-age *Z* score (IAZ) could be used in a similar way to the microbiota-for-age *Z* scores (MAZ) generated for Bangladeshi infants [16], helping to identify malnourished children most at risk of infection. As for MAZ, interpreting IAZ scores would require large-scale longitudinal assessment of immune development in healthy ‘exemplar’ children [16].

## Targeting Immunopathogenic Pathways in Malnutrition

Current nutritional interventions do not fully reverse morbidity or in undernutrition [8], but immune function is transiently improved following therapeutic feeding both in humans 11, 12, 79 and animals 41, 64, 73. The roles of defined dietary nutrients in immune priming and gut function 38, 49 support development of therapeutic foods to promote immune recovery in malnutrition (immunonutrition). Specific formulations have been developed for critically ill patients; however, meta-analyses and large-scale clinical trials are inconclusive on whether immunonutrition affects infections or mortality [83].

Few studies have directly targeted immune pathways in malnourished children; however, standard protocols for SAM treatment include antibiotics [84], which can reduce mortality and improve nutritional recovery [85]. The mechanisms through which antibiotics improve outcomes in malnutrition are unclear, but may include treating clinical and subclinical infections, reducing chronic inflammation or ameliorating enteropathy through changes in the microbiota [85]; antibiotic effects on immune function have not been evaluated. A complementary therapeutic approach would be to target inflammation using anti-inflammatory drugs. A promising pilot study of mesalazine in children with SAM and EED showed trends towards reduced markers of gut inflammation (fecal calprotectin) and microbial translocation (anti-LPS IgG) relative to placebo-treated controls [56]. Furthermore, several studies provide proof-of-principle that targeting immunopathogenesis in malnutrition is feasible. For example, 4 years of daily subcutaneous leptin injections reversed immune defects in obese children with congenital leptin deficiency [86]. A study of LPS challenge in healthy humans also demonstrated that *in vivo* administration of IFN-γ, and to a lesser extent GM-CSF, restored LPS-specific TNF-α responses [77], indicating that immunoparalysis can be safely and effectively reversed. Timing of interventions targeting immune dysfunction will likely be a crucial determinant of their efficacy. Although immunodeficiency presents during the first 1000 days, pre-conception interventions in mothers and fathers may be necessary to target the epigenetic origins of undernutrition 5, 32, 33, 34 and the associated predisposition to adult metabolic syndromes 4, 9.

## Concluding Remarks

It has long been apparent that infectious mortality is elevated in undernutrition and obesity (Box 1) and that immunodeficiency is a hallmark of malnutrition (Box 2). In this review we have summarized data demonstrating that immune dysfunction is not only a consequence of inadequate diet but also contributes directly to mortality and morbidity associated with malnutrition. Emerging data from animal models and human cohorts indicate that immune dysfunction underlies the etiology of malnutrition early in the life-course, through epigenetic modifications of infant immune genes; the influence of chronic inflammation on growth hormones, HPA signaling, adiposity and metabolism; altered gut structure and function; reduced immune-mediated protection from infections 63, 64, 79; and the interplay between environment, nutrition, and immune development (Figure 1). A causal role for immune dysfunction in human undernutrition has been postulated in light of associations between elevated inflammatory mediators at birth and subsequent stunting [14], as well as consistent indications that inflammation dysregulates growth hormones 14, 56, 58. Metabolic defects conferred via epigenetic modification of immune genes can be inherited by the adequately nourished offspring of malnourished fathers 36, 37 and from ancestors exposed to deficient diets several generations earlier [69]. More broadly, immune dysfunction in malnourished mothers has a causal impact on infant nutritional status because reduced transfer of protective maternal immune factors and increased exposure to pathogenic microbes and proinflammatory mediators confer an elevated metabolic cost on developing infants [20]. Few of the pathways linking malnutrition and immune dysfunction in murine experiments, *in vitro* studies, and human overnutrition have been well defined in undernutrition; we therefore propose a series of research questions that urgently need to be addressed in future studies (see Outstanding Questions). Better understanding the role of the immune system in malnutrition will inform targeted interventions for vulnerable children with undernutrition, where there is a crucial need for new approaches to reduce global mortality.

Outstanding Questions *Immune Development.* What is the trajectory of healthy immune development in early life, and how is this perturbed in malnutrition? Identifying the functional characteristics of ‘immune faltering’, and the age at which it emerges, will identify optimal timings for therapeutic interventions.

Is there a relationship between breast-milk components, infant immune function, and subsequent growth defects? Characterization of the PAMPs, immune mediators, and microbes delivered to infants with healthy immune development and growth patterns during exclusive breast-feeding may assist the design of immunonutrition for infants at risk of stunting.

How is the immunoepigenome at birth related to the parental immunoepigenome and subsequent immune function during early life?

*Gut Immune Responses.* Does expression of nutrient-sensing receptors and PRRs by human immune cells differ according to nutritional status? Is this evident systemically or restricted to mucosal sites?

What is the relationship between microbiota-for-age and immune function-for-age in healthy and malnourished infants? Given that the microbiota and immune system have reciprocal effects, it will also be important to determine whether this relationship changes in response to different therapeutic interventions (e.g., feeding, antibiotics, anti-inflammatory treatment).

Which mucosal cell types drive enteropathy in human EED? Is there a systemic immune biomarker of EED?

*Immunometabolism.* Is the immunometabolic profile in infant malnutrition related to immunopathology in adulthood?

Do adipokine and glucocorticoid hormone levels differ according to the severity of malnutrition? Are their levels related to immune function in the periphery, gut, or adipose tissue?

*Immune Priming and Memory.* Does undernutrition alter effector memory T cell function or trained immunity? Functional assays of immune priming and memory will be particularly pertinent to understanding the relationship between malnutrition and infectious mortality.

## Box 1 Epidemiology and Clinical Features of Malnutrition

Malnutrition is a clinical syndrome that encompasses a spectrum of anthropometric defects from wasting (low weight-for-height) and stunting (low height-for-age) in undernutrition to overweight (body mass index, BMI, of 25–30) and obesity (BMI >30) in overnutrition. An estimated 3.1 million children under 5 years die annually as a result of undernutrition, accounting for 45% of all child mortality [2]. The global prevalence of overweight in the under 5 year age group increased by 54% between 1990 and 2011 [2].

Growth faltering in developing countries is often evident at birth. Weight-for-age and length-for-age *Z* scores continue to decline over the first 2 years of life, with little recovery thereafter [70]. Maternal height and infant birthweight are consistent predictors of infant growth (short mothers bear small babies who become stunted adults themselves) [20]. Stunting affects around 165 million children worldwide (www.who.int/nutgrowthdb/estimates/en) and is associated with short-term increases in infectious mortality and long-term neurodevelopmental defects 20, 65, 87. Stunted children are shorter than healthy reference populations (height-for-age *Z* score ≤2) and may exhibit reduced exploratory behavior and physiological arousal [87], but are otherwise clinically well.

Wasting affects approximately 52 million children (www.who.int/nutgrowthdb/estimates/en) and has a stronger association with case mortality than stunting. Wasting is characterized by loss of fat and muscle in the thighs, buttocks, upper arms, and ribs; severe acute malnutrition (SAM) is characterized by severe wasting (mid-upper arm circumference (MUAC) <115 mm for children aged 6–59 months and/or weight-for-height *Z* score ≤3; www.who.int/childgrowth/standards). Kwashiorkor is a distinct SAM phenotype typified by skin and hair changes, irritability, symmetrical edema, and fatty liver infiltration. Children with uncomplicated SAM (no apparent infections and good appetite) can be managed in the community with energy-dense micronutrient-supplemented foods [84]. Children with complicated SAM (clinically unwell and/or refusing feeds) are often physiologically unstable, with impaired organ function, coinfections, micronutrient deficiencies, and a high risk of dying, which requires immediate hospitalization, supervised feeding and rehydration, and broad-spectrum antibiotics even if symptoms of infection are absent [84].

Early-life undernutrition confers an increased risk of obesity in adulthood 4, 9. This ‘double burden’ of malnutrition disproportionately affects low- and middle-income countries undergoing rapid socioeconomic changes [4]. Clinical symptoms of obesity include high percentage adipose tissue, high circulating levels of triglycerides, high blood pressure which can lead to cardiovascular disease, and high blood glucose levels reflecting insulin resistance which can lead to type-2 diabetes 4, 6, 9, 25. Similarly to undernutrition, overweight is associated with an increased risk of all-cause [1] and infectious mortality [25].

## Box 2 Immune Defects in Undernourished Children

A recent systematic literature review [23] identified 245 studies published between 1957 and 2014 describing immune parameters in undernourished children (age 0–5 years). However, the review highlights that the majority of studies were conducted several decades ago using out-dated immunology techniques and focused on hospitalized children with severe forms of malnutrition and multiple coinfections. Characterization of immunodeficiency was limited by a lack of longitudinal studies, particularly for mild and moderate malnutrition. The precise nature of immunodeficiency in undernutrition therefore remains uncertain; however, the consensus from the available evidence is that both innate and adaptive immunity are impaired by malnutrition. Defects in innate immune function include impaired epithelial barrier function of the skin and gut, reduced granulocyte microbicidal activity, fewer circulating dendritic cells, and reduced complement proteins, but preserved leukocyte numbers and acute phase response. Defects in adaptive immune function include reduced levels of soluble IgA in saliva and tears, lymphoid organ atrophy, reduced delayed-type hypersensitivity responses, fewer circulating B cells, a shift from Th1-associated to Th2-associated cytokines, and lymphocyte hyporesponsiveness to phytohemagglutinin, but preserved lymphocyte and immunoglobulin levels in peripheral blood. Despite this, most malnourished children seem to respond adequately to vaccination, although the timing, quality, and longevity of vaccine-specific responses may be impaired 71, 72.

There is an evident need for contemporary studies of childhood undernutrition incorporating up-to-date functional immunological methods in well-characterized longitudinal cohorts of children. Studies need to define malnutrition using current metrics of stunting, wasting, or both together, with appropriate well-nourished comparison groups, and evaluate associations between immune parameters and clinical outcomes. Development of novel experimental approaches to explore immune ontogeny 88, 89, epigenetics 5, 32, 33, 34, immunometabolomics [61], the gut microbiome [49] and virome [90], enteropathy [59], and nutrient-sensing 38, 39 also provide unprecedented opportunities for translation into immunological studies of childhood undernutrition. Because immunodeficiency is also a feature of overnutrition (reviewed recently 6, 25), immunological studies in overweight and obese children may also be pertinent to understanding immunopathogenesis in undernutrition.

## Box 3 Immune Development During the First 1000 Days of Life

The first 1000 days of life (from conception to 2 years) is identified as a developmental window of opportunity for therapeutic interventions for undernutrition (www.thousanddays.org/) and is also a critical period in immune development [91]. Evidence that early-life malnutrition confers life-long immunodeficiency 4, 20, 24 is consistent with a ‘layered’ model of immune ontogeny in which adult immune function relies on developmental steps at earlier ages (e.g., fetal and adult CD4^+^ T cells derive from phenotypically-distinct hematopoietic stem cells [89]). Immune responses are more tolerogenic in infancy than adulthood, fetal naïve T cells differentiate into Tregs more readily [89], Tregs comprise higher proportions of CD4^+^ T cells in pediatric (30–40%) versus adult tissues (1–10%) [88], and maternal alloantigen-specific T cell expansion is suppressed by high Treg frequencies in infant lymphoid organs [92].

GALT population by lymphocytes begins *in utero*, but postnatal cues from environmental (dietary and microbial) antigens instigate optimal positioning and accumulation of peripherally matured cells 38, 88. Analysis of infant organ donor tissues showed that activated T cell populations initially expand at mucosal sites, likely due to ingested or inhaled antigen exposure [88]. Naïve (CD45RA^+^CCR7^+^) T cells predominate in most infant tissues (70–90%) relative to adults (<50%); however, the gut and lungs had the highest T_EM_ (CD45RA − CCR7 − ) proportions in the infant tissues analyzed [88]. A role for immune receptor interactions with environmental antigens in murine gut development has been conclusively demonstrated 48, 93. Vascularization of the ileum, epithelial cell proliferation, and colonic mast cells are perturbed in neonatal mice lacking the TLR and IL-1 receptor-associated signaling molecules MyD88 and TRIF [48]. Mice weaned onto macromolecule-depleted chow devoid of dietary antigens lack peripherally generated Treg (pTreg) cells required for oral tolerance [93]. These effects were distinguishable from those of the microbiota because MyD88- and TRIF-deficient animals had similar fecal microbiota compositions to co-housed littermates [48]**.** Comparison of intestinal tissue from macromolecule-deficient mice, germ-free mice and mice exposed to a normal diet and microbial colonization showed that diet-induced and microbiota-induced pTreg populate distinct locations [93].

Infant diet shapes environmental antigen exposure, and weaning alters mucosal development [48]. Exclusive breastfeeding is recommended for the first 6 months of life in developing countries, thereby providing essential nutrients and maternal immune factors [91]. Early introduction of non-breast milk foods increases risk of diarrhea [94], which coincides with growth faltering 20, 55. Breast milk also contains microbes [95] which engage immune receptors and seed the gastrointestinal tract [96]. Birth mode (vaginal or caesarean) [96] and breastfeeding duration [95] shape the infant microbiota.

## Box 4 The Microbiota in Malnutrition

The microbiota plays a direct role in digestion by metabolizing nutrients that the human gut cannot 38, 49. Without signals from microbial components, mucosal immune development and digestion are markedly impaired 48, 93, 97, and reciprocal immune signals shape microbiota composition [97]. Unlike intestinal epithelial cells, which are segregated from the gut lumen by a mucus layer and innate defense mechanisms, the microbiota is in intimate contact with ingested food and environmental contaminants, and the emerging view is that the microbiota is a sensor of dietary change 11, 38, 49. This hypothesis has been confirmed through systematic modifications of dietary protein, fat, polysaccharide, and simple sugars in gnotobiotic mice populated with 10 defined human gut bacterial species, in which each nutrient governed distinct microbial compositions, and protein changes accounted for the largest variations [98].

16S ribosomal RNA sequencing of bacteria in human fecal samples from developing countries has highlighted the specific contribution of the microbiota to undernutrition in children 13, 16, 50, 90, and complementary murine models can be generated via fecal transplantation from children with SAM into gnotobiotic mice 13, 16, 90. Cohorts of twins and triplets discordant for malnutrition, and detailed longitudinal characterization of healthy microbial colonization in healthy ‘exemplar’ infants inhabiting similar environments, have allowed a range of common confounders in human malnutrition studies to be controlled for, including age-dependent microbial plasticity; environmental factors such as diet, pathogen exposure, and WASH; and host genetic variation.

Relative to age-matched adequately nourished children, children with SAM have defects in the diversity and composition of commensal microbes 13, 16, 90, termed ‘microbiota immaturity’ [16]. Although therapeutic feeding interventions ameliorate changes in the bacterial microbiota of children with SAM [16], the virome is unaffected [90], and the bacterial microbiome returns to its pre-intervention composition after feeding interventions have ceased 13, 16. Healthy fecal metabolite profiles in gnotobiotic mice transplanted with the microbiota from undernourished children were also restored when animals were fed nutrient-rich chow, but regressed once mice returned to a nutrient-poor ‘Malawian’ diet, reflecting the direct impact of microbial changes on digestion [13]. Microbiota composition and fecal and urinary metabolite profiles also shifted in rural-dwelling South Africans fed a high-fat, low-fiber ‘Westernized’ diet [12]. These observations demonstrate that microbiota composition and the quality of nutrient digestion are rapidly reconfigured by dietary interventions, but these changes are not sustained, either owing to underlying defects in gut immune function, reintroduction of an inadequate diet, or continued exposure to unfavorable environmental selection pressures (e.g., poor WASH).
